# How to Calculate Renyi Entropy from Heart Rate Variability, and Why it Matters for Detecting Cardiac Autonomic Neuropathy

**DOI:** 10.3389/fbioe.2014.00034

**Published:** 2014-09-09

**Authors:** David J. Cornforth, Mika P. Tarvainen, Herbert F. Jelinek

**Affiliations:** ^1^Applied Informatics Research Group, Faculty of Science and IT, The University of Newcastle, Callaghan, NSW, Australia; ^2^University of Eastern Finland, Kuopio, Finland; ^3^Kuopio University Hospital, Kuopio, Finland; ^4^Charles Sturt University, Albury, NSW, Australia

**Keywords:** Renyi entropy, heart rate variability, cardiac autonomic neuropathy, probability estimation, disease discrimination

## Abstract

Cardiac autonomic neuropathy (CAN) is a disease that involves nerve damage leading to an abnormal control of heart rate. An open question is to what extent this condition is detectable from heart rate variability (HRV), which provides information only on successive intervals between heart beats, yet is non-invasive and easy to obtain from a three-lead ECG recording. A variety of measures may be extracted from HRV, including time domain, frequency domain, and more complex non-linear measures. Among the latter, Renyi entropy has been proposed as a suitable measure that can be used to discriminate CAN from controls. However, all entropy methods require estimation of probabilities, and there are a number of ways in which this estimation can be made. In this work, we calculate Renyi entropy using several variations of the histogram method and a density method based on sequences of RR intervals. In all, we calculate Renyi entropy using nine methods and compare their effectiveness in separating the different classes of participants. We found that the histogram method using single RR intervals yields an entropy measure that is either incapable of discriminating CAN from controls, or that it provides little information that could not be gained from the SD of the RR intervals. In contrast, probabilities calculated using a density method based on sequences of RR intervals yield an entropy measure that provides good separation between groups of participants and provides information not available from the SD. The main contribution of this work is that different approaches to calculating probability may affect the success of detecting disease. Our results bring new clarity to the methods used to calculate the Renyi entropy in general, and in particular, to the successful detection of CAN.

## Introduction

Cardiovascular function is controlled by intrinsic and extrinsic mechanisms including membrane properties of the sino-atrial node, neuro-hormonal, and autonomic nervous system (ANS) modulation (Valensi et al., 2002; Vinik et al., 2003). The natural rhythm of the human heart is known to vary in response to sympathetic and parasympathetic signals and dysfunction of either of these mechanisms is often associated with diabetes, which can manifest at different times during diabetes disease progression. This dysfunction is referred to as cardiac autonomic neuropathy (CAN) (Pop-Busui, 2010; Tarvainen et al., 2013). As it affects the control of heart rate, it should be manifested in changes to cardiovascular rhythm, which are observable and therefore classifiable.

**Figure 1:**
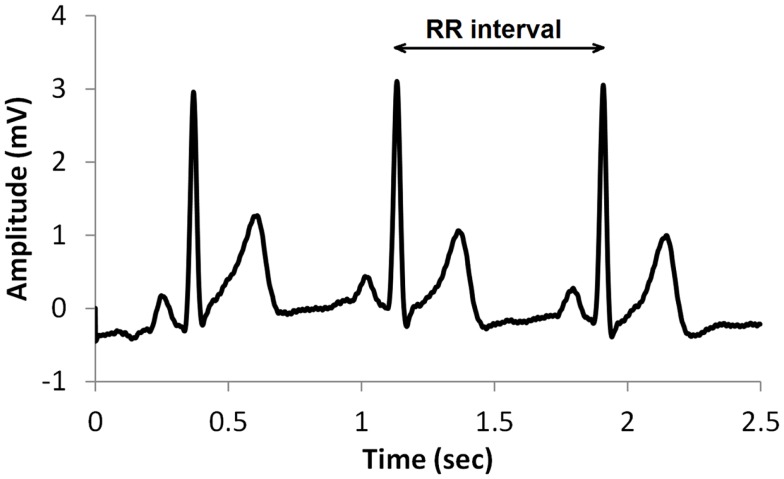
**A typical ECG signal showing the RR interval**.

A common type of ECG signal is shown in Figure 1. Such signals have been studied extensively and the diagnostic value of the different features is well established. The RR interval, shown in Figure 1, is the time between successive heart beats. Heart rate is the reciprocal of this. Generally, sympathetic activity increases heart rate and decreases variability, whereas parasympathetic activity decreases heart rate and increases variability (Berntson et al., 1997). Therefore, heart rate variability (HRV) is a useful indication of the health of the cardiovascular system, and is commonly used in assessing the regulation of cardiac autonomic function (Flynn et al., 2005; Vinik et al., 2013). HRV may be described by a variety of measures such as time domain, frequency domain, and non-linear measures (TFESC, 1996; Khandoker et al., 2009; Sacre et al., 2012).

Guidelines for the use of HRV for clinical practice were established in 1996 and included time and frequency domain measures as well as non-linear analysis and geometric methods from the time signal in the form of heart rate or inter-beat intervals (RR intervals) (TFESC, 1996). Geometrical methods are less sensitive to noise in the biosignal and offer a good alternative to the more often time and frequency domain analysis applied to determine HRV (Sztajzel, 2004) but require longer recording times (Malik and Camm, 1993). Geometric methods require the time series to be converted to a discrete scale, which allows the binning of RR intervals as histograms. More recently, geometric-based methods have also been shown to be robust for classification of CAN with shorter recording periods (Karmakar et al., 2009, 2013; Jelinek et al., 2013). Geometric methods are in general based on converting the RR intervals into a geometric form such as a triangle or ellipse, which can assist with visualization and can provide summary measures such as measures that describe the shape of such figures, to be calculated. Another approach is to express the RR intervals as a frequency distribution histogram, which can then be analyzed (Nasim et al., 2011). Common examples of HRV features derived from the geometric forms include the triangular index (Tri), triangular interpolation of NN interval (TINN), the Poincaré plot including the complex correlation method (CCM), and wavelet-based analysis (Tulppo et al., 1996; Acharya et al., 2002, 2006; Voss et al., 2007). More recent geometric methods for HRV analysis include the entropy measures, especially if the feature extracted is based on a histogram presentation of the RR intervals or autoregressive spectral estimation of the tachogram (Wessel et al., 2000; Tarvainen et al., 2013).

Non-linear measures include determination of the time signal’s entropy in an attempt to quantify randomness in the system and include approximate entropy (ApEn), sample entropy (SampEn), and multiscale entropy (MSE). Renyi entropy generalizes the Shannon entropy and includes the Shannon entropy as a special case (Rényi, 1960). Renyi entropy *H* is defined as:

(1) $$H\left(\alpha \right)=\frac{1}{1-\alpha }\log_{2}\left(\sum_{i=1}^{n}p_{i}^{\alpha }\right)$$

where *p_i_* is the probability that a random variable takes a given value out of *n* values, and α is the order of the entropy measure. *H*(0) is simply the logarithm of *n*. As α increases, the measures become more sensitive to the values occurring at higher probability and less to those occurring at lower probability, which provides a picture of the RR length distribution within a signal. However, the entropy requires an estimate of probabilities, and there are a number of ways in which this can be determined.

**Figure 2:**
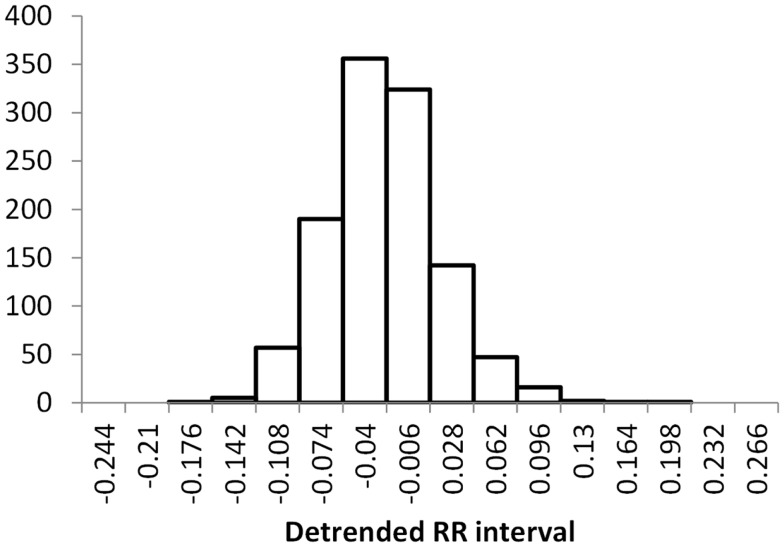
**Histograms of the detrended RR intervals for participants in the Normal group made using the same bin divisions for all data used**. The vertical axis shows the count of RR intervals falling into each bin. The SD is 0.050.

**Figure 3:**
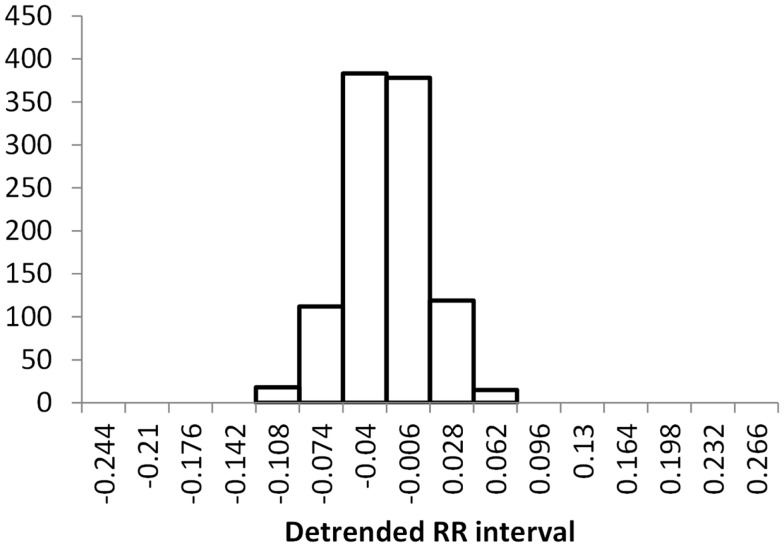
**Histograms of the detrended RR intervals for participants in the Early CAN group made using the same bin divisions for all data used**. The vertical axis shows the count of RR intervals falling into each bin. The SD is 0.038.

**Figure 4:**
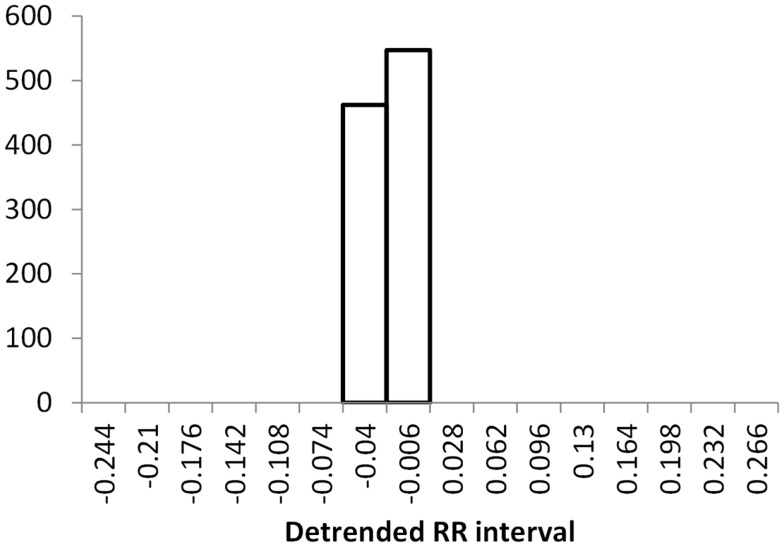
**Histograms of the detrended RR intervals for participants in the Definite CAN group made using the same bin divisions for all data used**. The vertical axis shows the count of RR intervals falling into each bin. The SD is 0.026.

The histogram method has advantages in terms of its low computational effort and its ability to allow a simple visualization of the distribution of RR values. However, its reliance on binning the data points introduces an artificial discretization, leading to a boundary problem where an individual value may be included in one bin or the other depending on a very small perturbation. This can be ameliorated by using a smoothing method that spreads data points into adjacent bins, but does not take into account how close a particular data point was to the bin boundary. Figures 2–4 show histograms of detrended RR intervals using data from participants in this study classified as Normal (N), Early CAN (E), or Definite CAN (D), respectively. The effect of the discretization is clear, especially for the data from participants with definite CAN (Figure 4), where all RR intervals fell into only two bins. The difference in distribution between the three classes is also clear, with participants in the Normal group (Figure 2) showing much greater variability of RR intervals than those with CAN (Oida et al., 1999; Khandoker et al., 2009). Notice also that this particular example of early CAN (Figure 3) appears to fit somewhere between Normal and Definite CAN.

An alternative approach is to estimate the probability of an individual RR interval using a notional density of space around the individual data point. This can be done by considering other RR intervals in the vicinity of the individual RR interval using Euclidean distance and imposing a threshold. For example, a calculation of sample entropy is described in Costa et al. (2002) as counting all RR intervals that are closer to the individual RR interval given a specific threshold. However, this still introduces a boundary problem and depends on choosing a suitable value for the threshold. Alternatively, a Gaussian kernel is centered on the individual RR interval and all RR intervals are added, weighted by the Gaussian function, based on the distance between the individual RR interval and the other RR intervals. A density measure can be calculated for the individual RR interval with index *i*, as the sum of all contributions from other RR intervals with index *j*:

(2) $$\rho_{i}=\frac{1}{\sigma \sqrt{2\pi }}\sum_{i=1}^{n}e^{-\frac{{\mathrm{dist}}_{ij}^{2}}{2\sigma^{2}}}$$

where σ is a parameter of the method and controls the dispersion of the function, and dist*_ij_* is the Euclidean distance between two RR intervals:

(3) $${\mathrm{dist}}_{ij}=\sqrt{{\mathrm{RR}}_{i}^{2}-{\mathrm{RR}}_{j}^{2}}$$

Apart from the advantage of allowing a continuous rather than a discretized measure, this method also allows more than one dimension, as long as a suitable distance measure can be provided. In the case of RR intervals, higher dimensions allow sequences of RR intervals to be compared, rather than individual RR intervals. In this case, a single RR interval with length RR_i_ is replaced by a sequence of RR intervals *S_i_*_,λ_ = {RR*_i_*_+1_, RR*_i_*_+2_, RR*_i_*_+λ_}, where λ is the number of intervals in the sequence of length λ. The calculation of distance now becomes:

(4) $${\mathrm{dist}}_{ij}=\sqrt{\sum_{k=1}^{\lambda }\left({\mathrm{RR}}_{i+k}^{2}-{\mathrm{RR}}_{j+k}^{2}\right)}$$

where 1 ≤ *k* ≤ λ.

This value of dist*_ij_* can be used in Eq. 2 to estimate the probability of a sequence of RR intervals, and the resulting probability can be used in Eq. 1 to calculate the Renyi entropy of a sequence of RR intervals. This provides several advantages over the histogram method. First, there are no boundary issues associated with placing RR intervals into a finite number of bins, where the bin boundaries are set at arbitrary values, possibly introducing bias. Second, this method can be applied to sequences of RR intervals, where this is infeasible for the histogram method. This method does, however, have two parameters to set: the sequence length λ and the Gaussian dispersion σ.

**Figure 5:**
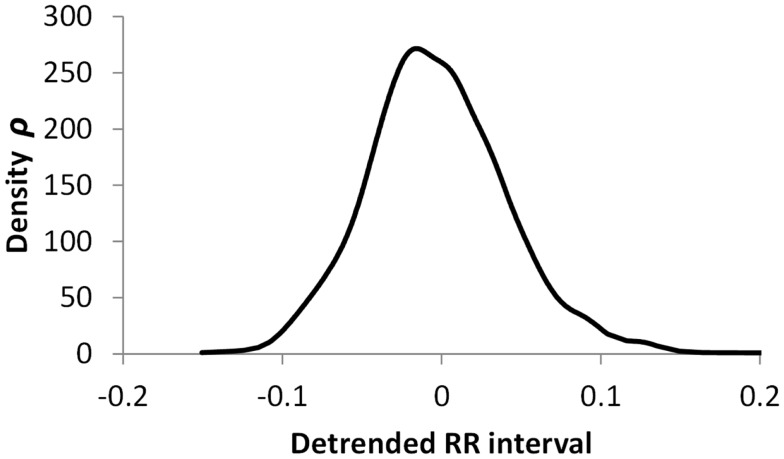
**Density of detrended RR intervals plotted against the RR interval value for participants in the Normal group**. The SD is 0.030.

**Figure 6:**
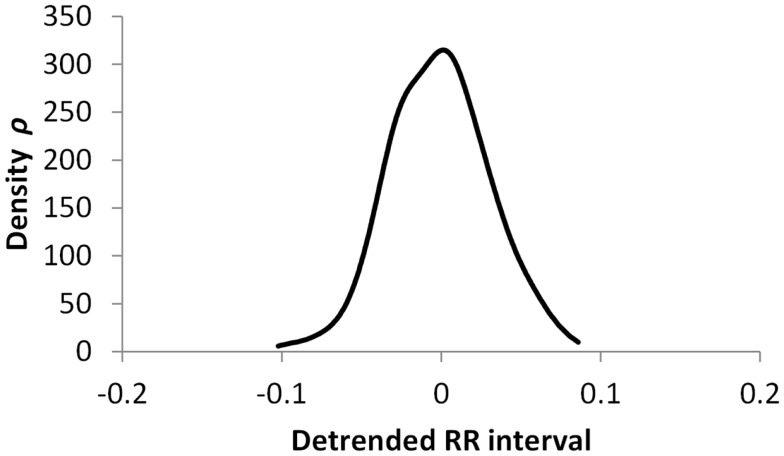
**Density of detrended RR intervals plotted against the RR interval value for participants in the Early CAN group**. The SD is 0.023.

**Figure 7:**
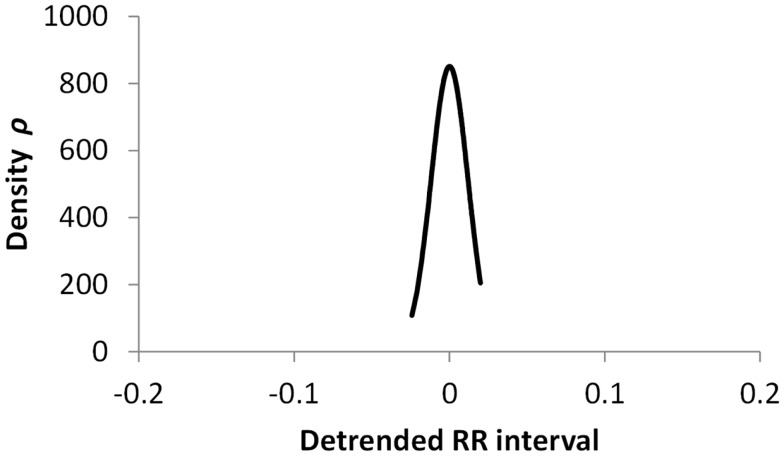
**Density of detrended RR intervals plotted against the RR interval value for participants in the Definite CAN group**. The SD is 0.006.

Figures 5–7 show visualization of the results of this process, for Normal, Early CAN, and Definite CAN classes, respectively. For every RR interval, its density measure ρ has been plotted against the RR interval value. This results in a much smoother curve than the histogram method, and note that each graph shown here comprises approximately 1000 points. As with the figures showing histograms, the effects of CAN may be seen in terms of the reduced variability of heart rate.

## Materials and Methods

The work reported here used data from the Charles Sturt Diabetes Complications Screening Group (DiScRi), Australia (Jelinek et al., 2006). The study was approved by the Charles Sturt University Human Ethics Committee, and written informed consent was obtained from all participants. A 20-min lead II ECG recording was taken from participants attending the clinic, using a Maclab Pro with Chart 7 software (ADInstruments, Sydney, NSW, Australia). Participants were comparable for age, gender, and heart rate, and after initial screening, those with heart disease, presence of a pacemaker, kidney disease, or polypharmacy (including multiple anti-arrhythmic medication) were excluded from the study. The same conditions were used for each participant. The status of CAN was defined using the Ewing battery criteria (Ewing et al., 1985; Javorka et al., 2008; Khandoker et al., 2009), and each participant was assigned as either without CAN (71 participants), early CAN (67 participants), or definite CAN (11 participants). From the 20-min recording, a 15 min segment was selected from the middle in order to remove start up artifacts and movement at the end of the recording. From this shorter recording, the RR intervals were extracted. No other information was used in this study. The RR interval series for each participant was pre-processed using the Kubios HRV software (Tarvainen et al., 2014). Ectopic and other aberrant beats, if any, were first corrected using a simple threshold based artifact correction algorithm of the software. The very low frequency trend components (frequencies below 0.04 Hz) were then removed from the RR interval series using a smoothness priors detrending method (Tarvainen et al., 2002). All the HRV measures were then applied on these pre-processed data.

The SD of RR intervals as a time domain feature and the Renyi entropy, using a scaling exponent α of integer values from −5 to +5, were analyzed. The Renyi entropy was calculated using nine different methods for calculating the probabilities, resulting in 90 Renyi measures for every participant (values for α = 0 were discarded since these measure only the support). For probabilities calculated using histograms, the RR intervals were separated into 30 bins using bin boundaries calculated in two different ways. In the first binning method, the maximum and minimum of all RR intervals in the entire datasets were calculated. The interval between the global maximum and minimum was divided into 30 equal portions, and a histogram was formed for each participant using these bin boundaries. Examples of this approach are shown in Figures 2–4. It may be observed that some of the histogram bins are effectively unused in this approach. In the second binning method, the maximum and minimum of the RR intervals for just one participant were used to calculate the boundaries of 30 bins in order to form a histogram for that participant. In this approach, all histograms have 30 useful bins.

After calculating each histogram by either method, the result is an estimate of the distribution of RR intervals for one participant in the study. To obtain probability values, each histogram was normalized by dividing the number of RR intervals in each bin by the total RR intervals in the sequence. This resulted in estimates of the probability of each bin, and these 30 probability values were used to calculate the Renyi entropy for the participant. This was repeated to calculate the Renyi entropy for each participant.

For each histogram, smoothing was performed using a five-term Gaussian kernel filter, and another set of Renyi entropy values was calculated from the smoothed histogram. The smoothing calculates a new frequency *m* for each bin *i* as follows:

(5) $$m_{i}^{*}=a_{2}m_{i-2}+a_{1}m_{i-1}+m_{i}+a_{1}m_{i+1}+a_{2}m_{i+2}$$

where *m_i_* is the number of samples falling into bin *i*, and $m_{i}^{*}$ is the filtered number of samples in bin *i*. The filter coefficients *a_k_* were calculated from a Gaussian function:

(6) $$a_{k}=e^{-\frac{{\left(i-k\right)}^{2}}{2\sigma^{2}}}$$

where σ is the range parameter of the filter. In this work, σ was set to 1, so the filter parameters used were 0.135, 0.607, 1, 0.607, and 0.135. As these parameters do not sum to 1, normalization is required, which was performed after calculating all probabilities, so that the sum of all 30 probabilities was made equal to 1. After Renyi values were obtained, these were also normalized by dividing by log_2_ of the number of bins. In this way, all Renyi entropies using α = 0 were 1, and could be compared between the different method used to calculate them, and allowed for the different number of intervals within each 15 min time series obtained from the participants.

For probabilities calculated using the Density method, probabilities were estimated from Eqs 2 and 4. Parameters were varied, as a range of sequence lengths λ were used, and the dispersion of the Gaussian function σ was varied in proportion, as detailed in the following list.

The nine methods are summarized here:

1. Histogram of 30 bins using a global maximum and minimum, based on the range of RR intervals obtained from all participants.
2. Same as method 1, but using Gaussian kernel smoothing.
3. Histogram using widths based on the individual maximum and minimum obtained from each participant.
4. Same as method 3, but using Gaussian kernel smoothing.
5. Density method for sequence length λ = 1 and σ = 0.01.
6. Density method for sequence length λ = 2 and σ = 0.02.
7. Density method for sequence length λ = 4 and σ = 0.04.
8. Density method for sequence length λ = 8 and σ = 0.08.
9. Density method for sequence length λ = 16 and σ = 0.16.

The Renyi measures were analyzed separately for each of the nine methods and positive and negative exponents were also treated separately in the analysis. For each measure, A Mann–Whitney test was performed to compare the Normal (N) to the Early (E) group, the Early to the Definite (D) group, and the Definite to the Normal group. Mann–Whitney tests were performed using the *u_test* procedure of *GNU Octave* with the default arguments. For each of the nine methods, we summarized the results of the separation between classes by finding the minimum *p*-value from three tests (Normal vs. Early, Early vs. Definite, and Definite vs. Normal) and for 10 different Renyi measures (−5 ≤ α ≤ +5). The minimum was used to indicate the best potential of each method to separate the classes and was followed by a more detailed examination or the actual *p*-values obtained. Thus the minimum *p*-value from 30 tests is used to summarize each method. The nine methods were also compared using the mean correlation coefficient from 11 Renyi values.

## Results

Table 1 summarizes the result of the nine versions of Renyi entropy used in the experiments. Each row corresponds to one of the methods of calculating probabilities. The numbers in column 1 correspond to those used in the Section “Materials and Methods.” The next two columns provide the minimum probability achieved by the Mann–Whitney tests comparing the three classes. These columns summarize the degree of class separation that was achieved. For example, method 3 produced Renyi entropy values that were unable to separate the three classes of participants, because the minimum *p-*values obtained from the Mann–Whitney test were 0.275 for the negative Renyi coefficients and 0.575 for the positive Renyi coefficients.

**Table 1:** **Summary of results of Mann–Whitney tests for Renyi entropy calculated using nine different methods of estimating probabilities**.

Method	Separation		Correlation		Verdict	
	Neg	Pos	Neg	Pos	Neg	Pos
1	0.043	0.000	0.109	0.910	Yes	No
2	0.126	0.000	0.074	1.061	No	No
3	0.275	0.575	0.106	0.003	No	No
4	0.129	0.680	0.115	0.005	No	No
5	0.106	0.000	0.027	0.850	No	No
6	0.014	0.000	0.067	1.025	Yes	No
7	0.008	0.000	0.106	1.019	Yes	No
8	0.003	0.000	0.209	1.043	Yes	No
9	0.000	0.000	0.467	0.915	Yes	No

Columns 4 and 5 provide the average Pearson *r^2^* value comparing the SD with the negative and for the positive Renyi values, respectively. For example, method 3 produced Renyi entropy values that were not correlated with the SD, with the mean Pearson *r^2^* value of 0.106 for the negative Renyi coefficients and 0.003 for the positive Renyi coefficients.

The final two columns provide a measure of the suitability of the method for assisting with the task of discriminating between classes. The verdict achieves an affirmative only if the probability value from columns 2 or 3 is <0.05 (a 5% probability that an apparent difference in classes occurs by chance), and if the correlation value from columns 4 or 5 is <0.5. For example, method 3 is considered unsuitable because although it produces measures that are uncorrelated with SD, the separation of classes was not achieved as indicated by the high *p*-values.

According to these criteria, the only measures that were deemed to satisfy the two outcome requirements are the negative Renyi measures for method 1 (Histogram) and all of the density methods except for method 5. However, Table 1 provides only a summary of results, as it lists only the minimum *p*-value found. Now, we examine more closely the results deemed satisfactory. All results are provided as *p-*values from the Mann–Whitney test on Renyi entropy calculated with negative exponents of α (in brackets) and significant results highlighted in yellow.

Table 2 shows the individual results using method 1. Only one test produced a *p-*value lower than 0.05, and that was using an exponent of −1, for discrimination between Normal and Early. The fact that method 1 did not distinguish between Normal and Definite casts doubt on this result, as it should be easier to detect a difference between control and definite CAN than a difference between control and early CAN. However, this may be a function of the smaller sample size for the definite CAN group or the shorter interval length. For all values of exponent, the correlation with the SD is small, suggesting that these Renyi measures contain additional information, with only 14.5% of the variability in the Renyi entropy explained by the SD.

**Table 2:** **Results of Mann–Whitney tests for Renyi entropy calculated with negative exponents and using method 1**.

Test	*H*(−5)	*H*(−4)	*H*(−3)	*H*(−2)	*H*(−1)
Normal vs. early	0.568	0.572	0.475	0.313	0.0427
Early vs. definite	0.388	0.360	0.388	0.403	0.332
Definite vs. normal	0.275	0.2625	0.250	0.204	0.0990
Pearson *r*^2^	0.0663	0.0683	0.0725	0.0846	0.145

The density measures provided better results in that they were able to differentiate between all three clinical groups and added additional information in terms of the differences of the biosignals between the three groups (Normal, early CAN, and definite CAN).

Using method 6 (sequences of length 2), only one test was significant, differentiating between Definite CAN and Normal with *H*(−1) (Table 3). Also, differentiation between Normal and Early CAN nearly reached significance (*p* = 0.0536). The result further suggests that 23.4% of the variability in Renyi entropy *H*(−1) is explained by the SD.

**Table 3:** **Results of Mann–Whitney tests for Renyi entropy calculated with negative exponents and using method 6**.

Test	*H*(−5)	*H*(−4)	*H*(−3)	*H*(−2)	*H*(−1)
Normal vs. early	0.950	0.945	0.826	0.559	0.0536
Early vs. definite	0.968	0.9148	0.872	0.726	0.230
Definite vs. normal	0.906	0.972	0.877	0.553	0.0136
Pearson *r*^2^	0.000458	0.00170	0.00566	0.0241	0.234

Table 4 shows the individual *p-*values from Renyi entropy calculated with negative exponents and using method 7 (sequences of length 4). Here, two tests produced a *p-*value lower than 0.05, implying that the Renyi entropy with exponent −1 may be able to discriminate these classes. The correlation between SD and Renyi entropy for *H*(−1) suggests that 26% of the variability of the latter is explained by the former.

**Table 4:** **Results of Mann–Whitney tests for Renyi entropy calculated with negative exponents and using method 7**.

Test	*H*(−5)	*H*(−4)	*H*(−3)	*H*(−2)	*H*(−1)
Normal vs. early	0.528	0.497	0.417	0.286	0.0314
Early vs. definite	0.319	0.306	0.269	0.201	0.1427
Definite vs. normal	0.0731	0.0694	0.0750	0.0591	0.00771
Pearson *r*^2^	0.0241	0.0294	0.0399	0.0690	0.260

The results for using method 8 (sequences of length 8) are shown in Table 5. The longer sequence length and larger Gaussian smoothing may have contributed to the entropies calculated with the more negative α values to also differentiate between Normal and Definite CAN. However, this was not the case for Normal versus early CAN. The contribution of the SD observed was found to be 11.8% for *H*(−5) and increased to 31% for *H*(−1) with a concomitant improvement in discriminatory power.

**Table 5:** **Results of Mann–Whitney tests for Renyi entropy calculated with negative exponents and using method 8**.

Test	*H*(−5)	*H*(−4)	*H*(−3)	*H*(−2)	*H*(−1)
Normal vs. early	0.104	0.101	0.0865	0.0566	0.00858
Early vs. definite	0.135	0.128	0.121	0.115	0.0908
Definite vs. normal	0.00657	0.00657	0.00548	0.00568	0.00278
Pearson *r*^2^	0.118	0.122	0.130	0.154	0.310

Method 9 (sequences of length 16) discriminated between Normal and Definite CAN as well as Normal versus Early CAN for all values of the exponent (Table 6), and also between Early and Definite CAN with *H*(−1). This suggests that increasing the sequence length allows further measures to be extracted that could be used in the discrimination of these disease subtypes. However, the correlation values are higher than those observed for shorter lengths (λ) and smaller Gaussian kernel smoothing with 43% of the variance explained by the SD for discrimination between any of the groups with *H*(−1).

**Table 6:** **Results of Mann–Whitney tests for Renyi entropy calculated with negative exponents and using method 9**.

Test	*H*(−5)	*H*(−4)	*H*(−3)	*H*(−2)	*H*(−1)
Normal vs. early	0.00105	0.00103	0.000854	0.000517	0.000115
Early vs. definite	0.062965	0.059243	0.06108	0.05073	0.0356
Definite vs. normal	0.00119	0.00110	0.000928	0.000816	0.00037
Pearson *r*^2^	0.353	0.353	0.357	0.373	0.434

**Figure 8:**
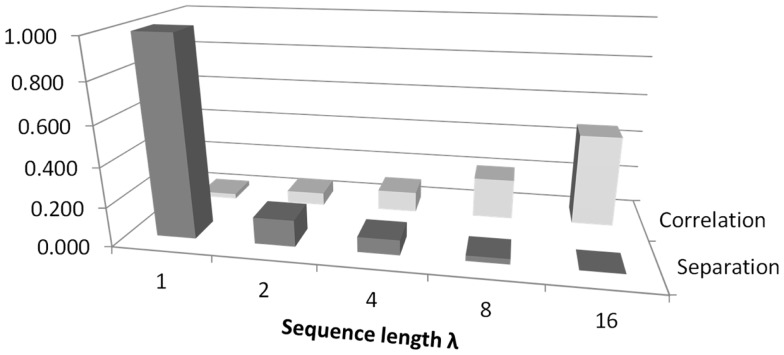
**An illustration of the trade-off between correlation and separation for choosing sequence length λ when using the density method of estimating probabilities for calculating Renyi entropy**. The figure relates to methods 5–9 as defined in the Section “Introduction.” The vertical axis shows Pearson’s *r*^2^ for correlation. For separation, the vertical axis is scaled for convenience, so that 1.0 corresponds to a *p-*value of 0.1.

Figure 8 illustrates the trade-off between separation and correlation for the density method. The horizontal axis shows the sequence length λ for methods 5–9 defined in the Section “Introduction.” In this figure, the average *p*-value obtained from *t*-tests is shown by dark gray bars and labeled as separation. The height of the bars has been scaled by 10, to lie in the range 0–1. The selected threshold of *p* = 0.05 therefore occurs at 0.5 on the vertical axis. All choices of sequence length λ >1 yield *p* < 0.05. Correlation is shown by the light gray bars, where the vertical scale indicates Pearson *r*^2^. The diagram shows that as the sequence length λ that is used to calculate probabilities increases, the ability to distinguish between classes improves. However, at the same time, the correlation between Renyi entropy and SD increases, suggesting that the Renyi entropy provides a diminishing about of useful information over and above that, which could be obtained from SD.

## Discussion

In this paper, we have shown that the Renyi exponent combined with the sequence length λ and the dispersion of the Gaussian function σ are important for classification paradigms such as determination of CAN progression. The main focus is to point out that any method for determining biosignal characteristics, such as entropy, needs to be able to show additional information about the biosignal, which is being analyzed. In the case of entropy evaluation, the standard method depends on binning the RR intervals into groups, and calculations are then based on the obtained histogram. Therefore, current entropy measures show a strong correlation with the SD of the biosignal, and in actuality, do not provide additional information.

Nine methods for calculating Renyi entropy were assessed in two ways: by the ability of the resulting values to allow identification of Definite CAN, Early CAN, or Normal, and whether the resulting values added extra information to that afforded by the SD. Results show that out of the histogram methods only the first method, which applied a histogram of 30 bins using a global maximum and minimum based on the range of RR intervals obtained from all participants, produced a positive assessment by these criteria. However, this method was only able to differentiate Normal and Early CAN using Renyi entropy with exponent α = −1. The positive Renyi coefficients associated with the remaining histogram methods were able to distinguish classes, but were also highly correlated with the SD and add no additional information.

The variants of the density method (methods 6–9) provided good separation between classes, but only for Renyi entropy calculated using negative exponents. The results using the density methods also improved as the sequence length was increased, but this also led to an increasing correlation with the SD. Therefore, an optimum sequence length that provides a compromise between good separation for classes and low correlation with SD is necessary. The current results suggest that for discriminating between Normal and early CAN, an optimal sequence length would be λ = 16 and a Gaussian kernel smoothing, σ = 0.16 with α being set at −5. The most difficult pair of classes to separate was Early CAN with Definite CAN. However, this is the problem likely to be of least interest. It is far more likely that a diagnostic test would rely on discrimination between Normal and Definite or between Normal and Early CAN (Vinik et al., 2013).

In order to distinguish between Normal and Definite classes, Table 5 represents a good compromise and corresponds to sequence length λ = 8 and σ = 0.08. The same situation also provides a good compromise for distinguishing Normal from Early CAN with *p* = 0.00858 and a correlation of only 0.310.

## Conclusion

The main finding of this work is that when calculating the Renyi entropy in order to provide variables that can discriminate between classes of CAN, the method chosen for estimation of probabilities may result in variables that are strongly correlated with the SD. As the latter is computationally easy to calculate, it casts doubt on why a more complicated measure such as entropy would be calculated, since it seems to provide little extra information. However, this work shows that more advanced methods of calculating probabilities can be applied, and that these yield entropy measures that not only provide good discrimination, but also contain information over and above that provided by the SD. The application of a variety of sequence lengths in the calculation of probabilities allows a trade-off between discrimination power and independence from SD. Use of a density method, based on sequences of RR intervals, produced Renyi measures that allowed discrimination between definite CAN, normal controls, and even early CAN. We found the best result for longer sequences of 8 or 16 beats. This suggests that a more sophisticated method for estimation of probabilities is justified by its improved results, and shows that Renyi entropy is suitable for use in discriminating CAN. Furthermore, we show that the method chosen for probability estimation is important and has a large effect on the outcome.

## Conflict of Interest Statement

The authors declare that the research was conducted in the absence of any commercial or financial relationships that could be construed as a potential conflict of interest.
